# The Attitude of Medical Students Toward Voluntary Body Donation: A Single Institute Survey and Narrative Review of Global Trends

**DOI:** 10.7759/cureus.40775

**Published:** 2023-06-22

**Authors:** Arthi Ganapathy, Praisy Joy, Sipra Rout, Manisha Gaikwad

**Affiliations:** 1 Anatomy, All India Institute of Medical Sciences, New Delhi, IND; 2 Anatomy, All India Institute of Medical Sciences, Bhubaneswar, IND

**Keywords:** religion, facilitator, barriers, undergraduates, practice, knowledge, voluntary body donation

## Abstract

Introduction: Voluntary body donation (VBD) programs form the backbone of cadaveric teaching and learning in medical schools. It benefits the medical fraternity the most. Yet, there is a dearth of VBD practice among medical students. We aimed to understand the knowledge and attitude of first-year medical students in a tertiary institute with a systematic review of willingness toward VBD among undergraduate students worldwide.

Methods: The first-year medical undergraduates were given a 12-item questionnaire to assess their knowledge and attitude toward VBD. Statistical tests were applied to analyze the difference between the variables. We systematically searched Google Scholar, PubMed, and SCOPUS databases until October 15, 2022. Data concerning knowledge, awareness, and attitude toward VBD among undergraduates of medical backgrounds were extracted and analyzed qualitatively.

Results: A total of 82.5% of students returned the completed responses. A significant association was seen between their attitudes toward whole body donation by strangers (p=0.043) and family members (p=0.035). The religion of the participants significantly affected their opinions on VBD and their willingness to pledge themselves (p=0.034). For the review, 20 studies were selected to be analyzed qualitatively. These studies included 4232 undergraduate students globally who were assessed for knowledge, awareness, and attitude toward VBD. Around 50% of the studies were published in India. The first study included was published in 2008. Seven studies were exclusively conducted on medical undergraduates, while the rest consisted of a mixed cohort. The attitude and knowledge of medical undergraduates on VBD were assessed via questionnaires containing both open-ended and closed-ended questions.

Conclusion: Based on observations from our survey and review, we concluded that while undergraduate students have a highly positive attitude toward VBD, their rate of pledging is low. The most prominent barrier to this attitude is their experience with cadavers in the dissection hall. Hence, we recommend a revisit of cadaver handling practices and the establishment of appropriate protocols for safe and deferential cadaver handling.

## Introduction

Current technologies and multimedia techniques are gradually shifting contemporary anatomy teaching and learning methods toward an interactive module with less time focused on dissection [1,2]. These technologies include extensive use of audiovisual aids such as PowerPoint presentations (Microsoft Corp., Redmond, WA, USA), 3D animations, e-learning access via video designs, etc. [3,4]. The National Library of Medicine has created the Visible Human Project, a detailed anatomical representation of the human male and female body that is available as open source [5] and can be used as a reference aid for anatomy teaching. Another learning tool designed for practical/dissection-oriented learning is the virtual dissection table. It has enhanced touch interaction [6,7]. A comparative evaluation of the use of virtual dissection tables and cadaveric dissection in previous studies has concluded that these tables can be used in adjunct to classical dissection techniques for a better understanding of cross-sectional anatomy. The main drawback of using virtual dissection tables is that we will never encounter any anatomical variations, which is the uniqueness and challenge of cadaveric dissection [8]. Synthetic cadavers are superior-quality human simulation models that give a visual and tactile experience when learning anatomy [9]. Even with extensive use of technology, there is a common consensus that these advancements can accentuate the dissection model of anatomy learning but can never replace it [10-13].

Anatomy without dissection is like a boat without an oar. Dissection forms the most vital part of teaching and learning anatomy, as the basis of anatomy is learning as a three-dimensional concept [14]. It gives a visual understanding with long-term retention and enhances the learner's psychomotor domain of Bloom’s taxonomy [15]. Autopsies also help discover anatomical variations that are necessary to avoid errors that occur during surgical treatment and to ensure proper management [16]. Dissection also enhances the development of compassion and provides human touch for developing empathy [17,18]. Many countries that initially replaced dissection with other technology later included it in their curricula, particularly for this reason [19].

The vitality of human cadavers as the pillars of safe medical and surgical practice is apparent, yet a shift towards alternate methods is rising over time. The prime reason is the shortage and non-availability of cadavers for dissection. The primary source of bodies for dissection is through voluntary body donation (VBD) programs and the procurement of unclaimed bodies [20]. Voluntary body donation is the selfless act of giving one's whole body after death for medical research and education [21,22]. Due to the high costs of virtual dissection tables and the growing number of medical universities in developing nations like India, VBD and the acquisition of unclaimed bodies continue to be the primary sources for studying anatomy [23].

Several initiatives are being taken by medical institutions globally to create body and organ donation programs [24-26]. The medical institutions work closely with various non-governmental organizations to create awareness and promote VBD among the general public [27]. Worldwide donors get high respect and honor as it is considered a sacrificial act for science. Body donors' families are felicitated in the gratitude ceremony [28,29]. Innovative awareness programs and the use of mass media to promote VBD are a few measures being implemented to address the scarcity of cadavers for dissection [30,31].

Even with these dynamic initiatives, the supply still needs to meet the demand. Studies have tried to analyze the barriers to VBD in general [31,32]. The VBD program benefits the medical fraternity the most. However, their willingness to use VBD for dissection is low [33,34]. The basis of anatomical knowledge for a first-year medical student is greatly enhanced by cadaveric dissection. This defines the significance of knowing how cadavers are procured for dissection. Therefore, we sought to comprehend first-year medical students' knowledge and attitude toward VBD by analyzing the facilitators and barriers faced by medical students globally regarding their willingness toward VBD.

## Materials and methods

Method for the original study

This study was approved by the Institutional Ethics Committee of the All India Institute of Medical Sciences, Bhubaneswar, India (approval no. T/IM-NF/Anatomy/22/85). The survey questionnaire was developed following a thorough examination of relevant literature on current attitudes and knowledge of VBD among medical students. It underwent validation by two subject experts separately. Subsequently, to assess the reliability of the questionnaire, it was administered to a small group of students to assess their comprehension of the questions. Any questions that posed difficulty for the participants were rephrased accordingly. Suggestions for possible improvements were considered, and further revisions were made to the questionnaire.

The questionnaire was administered through Google Forms (Google LLC, Mountain View, CA, USA) after receiving informed voluntary consent from the participants (see Appendix A). The first part of the questionnaire is related to the purpose of the assessment. The second part contains details about the participants, such as their names, ages, gender, and religion. This is followed by four questions in a dichotomous and semi-closed-ended format that assess the knowledge of VBD among first-year MBBS students. The questionnaire concludes with five questions that are a combination of the Likert scale, semi-closed-ended, and open-ended design to understand the attitude of first-year medical undergraduates toward VBD.

Statistical Analysis

To conduct the survey, convenience sampling was done based on the number of first-year MBBS students in the 2022 batch at our institute. The responses to each Likert scale question and other variables were summarized and described as numbers and percentages. All the decimals were rounded off to their nearest tenths or nearest whole numbers. The chi-square test was used to assess the differences among the different variables. A p-value of <0.05 was considered significant. Qualitative data obtained from responses to these semi-closed-ended questions were examined using an inductive qualitative method that involves open coding to discover emergent themes.

Method for narrative review

Objectives

This study aimed to evaluate the level of knowledge of VBD among undergraduates of medical backgrounds, assess the level of awareness and attitude toward VBD among medical undergraduates, and discover the facilitators and barriers toward the willingness to participate in VBD among medical graduates across the globe.

Protocol

The Preferred Reporting Items for Systematic Reviews and Meta-Analyses (PRISMA) protocol was adopted. See Appendix B for the characteristics of the final 20 articles included in this study.

The review tried to survey the attitude of medical students worldwide toward VBD and recognize various factors in favor of or against it. This is a barrier and facilitator type of review to identify factors affecting the knowledge and attitude of medical graduates regarding VBD. The inclusion and exclusion criteria applied in the systematic review are given in Table 1.

**Table 1:** Inclusion and exclusion criteria for articles

Variables	Inclusion criteria	Exclusion criteria
Population	Undergraduate medical students, nursing students, paramedical students	Healthcare workers of any age, medical doctors, general public
Intervention	Knowledge, attitude, perception	None
Comparison	None	None
Outcome	Various factors (facilitators, barriers)	

Search Strategy

The medical subject headings (MeSH) terms used in the search were: "Medical graduates, medical students, MBBS students, nursing students, paramedical students, voluntary body donation, body donation, self-body donation," "knowledge," "perception," and "awareness." These keywords were combined with Boolean operators (OR, AND). We did a comprehensive literature search using Google Scholar (38,200 articles), PubMed (175), and Scopus (27). We limited the articles to those published in the English language only. The articles that were common to all three databases were considered single. We conducted a thorough search of titles and abstracts. Articles that surveyed the perception of VBD among healthcare workers, medical doctors, or the general public only were excluded completely. Articles that were available in both were considered one. The records that were finally identified as eligible totaled 67. We screened the full text of these 67 articles and filtered 20 articles that met our criteria for the perception of various undergraduate students.

Data Extraction

Two independent reviewers gathered the data using a standardized form. Details of study design, year of publication, year of medical graduate, baseline, male:female ratio, age of students, sample size, place of study, the religion of the students, knowledge, awareness, and attitude toward VBD, and factors (barriers, facilitators) influencing VBD were recorded in a Microsoft Excel sheet (Microsoft Corp., Redmond, WA, USA). Outcome assessments were recorded in duplicate.

## Results

Original study

The Google forms were given to 120 students. Among them, 99 students returned the completed responses. There were 92 (92.93%) students in the age group of 17 to 19 years and 7 (7.07%) students in the age group of 20 to 23 years. Out of which, 25 (25.5%) students were females, and 74 (74.75%) were males. Analysis of the frequency distribution of students from different religions showed that 91 (91.92%) students were Hindus, 2 (2.02%) were Christians, 3 (3.03%) were Muslims, and 3 (3.03%) were atheists. The students' responses were analyzed, and the results are displayed in Table 2. A significant association was seen between responses to the question on attitudes toward whole-body donation by strangers. To this question, 85.71% of students who know someone who pledged to VBD responded positively (p=0.043, Table 2). A similar response was also found for the whole-body donation by a family member (p=0.035, Table 2). For the question on attitude toward whole-body donation by self, a significant difference in responses was observed based on the religion of study participants (p=0.034, Table 2). One volunteer expressed their opinion by disagreeing with the idea of donating their body, stating that organ donation is a superior choice because it can be utilized to save lives. As there were no other open responses to semi-closed-ended questions, thematic analysis was not done.

**Table 2:** Responses to the questionnaire by the undergraduate students based on various criteria *p-value is significant (<0.05) VBD: Voluntary body donation, KP: Known person, NKP: Not a known person

Questions	Response	Age		Gender		Religion				Knew a person who pledged for VBD	
		Group 1: 17 to 19 years	Group 2: 20 to 23 years	Female	Male	Hindu	Christian	Muslim	Atheist	KP	NKP
Are you aware of VBD?	Yes	82 (89.13%)	6 (85.71%)	21 (84%)	67 (90.54%)	80 (87.91%)	2 (100%)	3 (100%)	3 (100%)	12 (85.71%)	76 (89.41%)
	No	10 (10.86%)	1 (14.28%)	4 (16%)	7 (9.45%)	11 (12.08%)				2 (14.28%)	9 (10.58%)
	p-value	0.574		0.286		1.000				0.483	
Are you aware of the purpose of VBD?	Yes	88 (95.96%)	6 (85.71%)	23 (92%)	71 (95.94%)	86 (94.51%)	2 (100%)	3 (100%)	3 (100%)	13 (92.86%)	81 (95.29%)
	No	4 (4.34%)	1 (14.28%)	2 (8%)	3 (4.05%)	5 (5.05%)				1 (7.14%)	4 (4.71%)
	p-value	0.312		0.290		1.000				0.541	
Do you know the process of pledging for VBD?	Yes	80 (86.96%)	7 (100%)	23 (92%)	64 (86.49%)	79 (86.81%)	2 (100%)	3 (100%)	3 (100%)	12 (85.71%)	75 (88.24%)
	No	12 (13.04%)		2 (8%)	10 (13.51%)	12 (13.19%)				2 (14.29%)	10 (11.76%)
	p-value	0.593		0.7 25		1.000				0.677	
Attitude towards whole body donation to medical science by a stranger	Agree	54 (58.70%)	5 (71.43%)	17 (68%)	42 (56.76%)	54 (59.34%)	2 (100%)	2 (66.67%)	3 (100%)	12 (85.71%)	47 (55.29%)
	Neutral	34 (36.96%)	1 (14.29%)	6 (24%)	29 (39.19%)	32 (35.16%)		1 (33.33%)		1 (7.14%)	34 (40%)
	Disagree	4 (4.45%)	1 (14.29%)	2 (8%)	3 (4.05%)	2 (8%)				1 (7.14%)	4 (4.71%)
	p-value	0.217		0.273		0.453				0.043^*^	
Attitude toward whole-body donation to medical science by a family member	Agree	47 (51.09%)	5 (71.43%)	15 (60%)	37 (50%)	48 (52.71%)		2 (66.67%)	2 (66.67%)	12 (85.71%)	40 (47.06%)
	Neutral	34 (36.96%)	2 (28.57%)	7 (28%)	29 (39.19%)	33 (36.26%)	1 (50%)	1 (33.33%)	1 (33.33%)	2 (14.29%)	34 (40%)
	Disagree	11 (11.96%)		3 (12%)	8 (10.81%)	10 (10.99%)	1 (50%)				11 (12.94%)
	p-value	0.628		0.602		0.616				0.035^*^	
Attitude toward whole-body donation to medical science by self	Agree	61 (66.30%)	5 (71.43%)	20 (80%)	46 (62.16%)	61 (67.03%)		2 (66.67%)	3 (100%)	13 (92.86%)	53 (62.35%)
	Neutral	24 (26.09%)	2 (28.57%)	4 (16%)	22 (29.73%)	25 (27.47%)		1 (33.33%)		1 (7.14%)	25 (29.41%)
	Disagree	7 (7.61%)		1 (25%)	6( 8.11%)	5 (5.49%)	2 (100%)				7 (8.24%)
	p-value	1.000		0.330		0.034*				0.127	

Narrative Review

Characteristics of the Studies Included

After removing duplicates, 143 articles were identified in the initial screening according to the search strategy. Out of those, 76 papers dealing only with organ or blood donation were eliminated based on the title, abstract, and pertinent research question. Fifty articles were excluded where the study population was only healthcare personnel or the public. Twenty articles met the criteria we included for qualitative analysis.

These 20 articles were selected after full-text screening for undergraduate students' perception of VBD. These articles included undergraduates of medical backgrounds like MBBS students, nursing students, and paramedical students. Some of these studies have included healthcare staff like junior doctors, postgraduate students, technicians, even the public, engineering students, university students of mathematics along with undergraduate students of medical background [35-44]. We limited our analysis to the perception of undergraduate students in these studies. Among the 20, seven articles were identified with an exclusive study conducted on undergraduate students, including interns [45-51]. The undergraduate students' knowledge, attitude, and practice of VBD in all the articles mentioned above were included irrespective of their exposure to cadaveric dissection.

**Figure 1:**
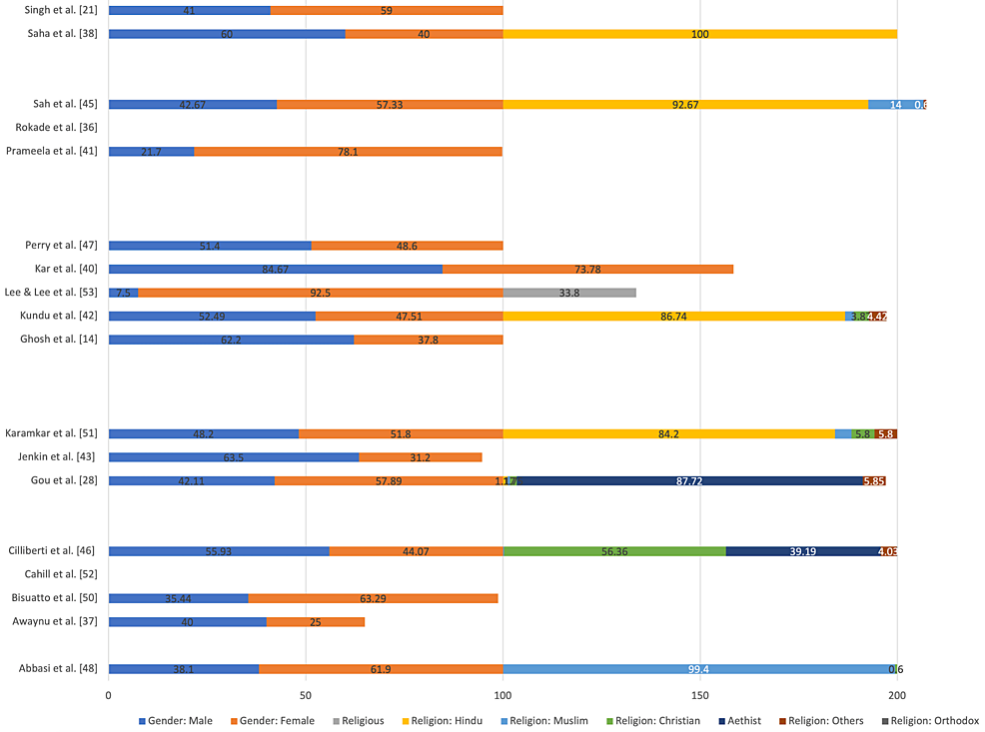
Gender and religious characteristics of the study cohort included in the systematic review represented as a percentage distribution

All these studies were carried out as cross-sectional studies with the administration of standardized and anonymous questionnaires, with one part dealing with demographic details (age, gender, ethnicity, nationality, religious belief, identified gender (if any), language spoken at home) of the participant. In contrast, other parts concentrated on the various aspects of knowledge, awareness, attitude, the willingness of the participant toward voluntary body donation, and attitude towards overall VBD (self, stranger, relatives). The characteristics of the study cohort (age, religion, gender distribution) are given in Table 3 and Figure 1. The questionnaires administered constituted open-ended and closed-ended questions for the participants to reflect on various parameters.

**Table 3:** Characteristics of the study cohort included in the systematic review (*) Samples included in the qualitative analysis

Authors	Sample size of cohorts included (total n=4232)	Age of the study cohorts (in years)
Cahil et al. [52]	N=212- first-year students^*^	18 to 24
Perry et al. [47]	N= 40, first-year graduate*	<21: 7.9%; 22 to 25:52.6%; 26 to 29: 34.2%; >30: 5.3%
Rokade et al. [36]	N=115 medical students*, n=110 working doctors, n=400 people from the general population of rural and urban areas.	18 to 25: 42.72%
Anyanwu et al. [37]	N=780 were students*, n=420 professionals	-
Saha et al. [38]	N=100 medical (MBBS) students*, n=100 engineering students, n=100 doctors	18 to 22
Mwachaka et al. [39]	N=150 undergraduate*, n=55 postgraduate	-
Abbas Asl et al. [48]	N=331 undergraduate students*	17 to 24: 97.6%; 24 to 30: 2.4%
Maitreyee Kar et al. [40]	N=227 (69=MBBS 1^st ^yr, 75= interns) *, n=144 (38 senior doctors, 35 nursing staff, 10 medical technicians)	-
Prameela et al. [41]	N=500 medical students* (undergraduates, graduates, and postgraduates)	-
Ghosh et al. [49]	N=100, first-year undergraduates*	18 to 23
Ciliberti et al. [46]	N = 1781, undergraduates*	<21:40.04%; 22 to 23:35.38%, >24: 24.58%
Sah et al. [45]	N=100 MBBS students*, n=100 paramedical students, n=100 nursing students	17 to 23: 82.67%, 24 to 30: 15%; 31 to 37: 2.3%
Biasiutto et al. [50]	N= 237 first-year MBBS*	-
Karmakar et al. [51]	N=361 undergraduates, MBBS*	<20:36.8%; 21 to 25:54%; 26 to 30:7.8%; >30: 1.5%
Varalakshmi et al. [44]	75 MBBS students*, 75 final-year engineering students	-
Guo et al. [28]	N=171, 1st and 3rd year medical students*	20.6 mean age
Kundu et al. [42]	N=181, MBBS students*, 449 paramedical staff	17 to 25
Lee et al. [53]	N=80, nursing students*	-
Singh et al. [21]	N= 400, medical and nursing students*	25 mean age
Jenkin et al. [43]	Anatomy experience (n=172)*, mathematics (n=133)	20 mean age

Seven out of these 20 studies conducted a pilot study on previous batches of students or random medicos, postgraduate students to assess the difficulty and clarity of the questions and to assess the time of response [39,42,43,45,48,50,52]. A team of subject experts later validated the questionnaires in the above studies before being administered to the target group. Two of these studies tested the reliability of the questionnaire based on Cronbach’s α score [43,53].

## Discussion

The present original study was conducted on undergraduate medical students on their entry into a tertiary healthcare institute. The age of the students ranged from 17 to 23 years. Saha et al. have also done their study on medical students in the age group of 18 to 22 years [38]. The questionnaire we gave the students was subdivided to understand their knowledge and attitude regarding VBD. Most of the students (87%) were aware of the process and purpose of VBD. These findings were similar to those reported by Kundu et al. (91.6%) and Singh et al. (90%) [42,21]. In the present study, their responses did not significantly differ according to age group, gender, religion, or if they were acquainted with a person who is a known donor. When analyzing the students' attitude toward VBD, a significant positive attitude and appreciation were seen towards donation by strangers (p=0.043) and family members (p=0.034) by the students who did not have a known person who pledged VBD. Such an association was not evaluated in the previous studies. This difference could be attributed to possible unpleasant experiences from a known person's body donation resulting in apprehension towards the act. Attitudes toward donation by self significantly differed based on religion (p=0.035). Atheists and most of the students who practiced Hinduism showed a positive attitude toward VBD when it came to pledging themselves when compared to those who practiced Islam and Christianity. Rokade et al. in their study, showed that according to their hospital data, most of the body donors were Hindus [36]. Like our study, they have affirmed the dominance of Hinduism in the VBD population. The other reasons they have attributed are the lower literacy rate and the more substantial effect of religion in other non-Hindu communities for the dismissive attitude toward VBD.

Knowledge of undergraduate medical students on VBD

Among the medical schools in 68 countries where human bodies are used for anatomy learning, only 32% of medical colleges use body donation as a source of cadaver resources. In the rest of the countries, it is primarily unclaimed bodies [20]. Looking at the global trends, Abbasi et al., in their study on 238 Iranian medical students, revealed that >70% were aware of VBD [48]. A study on the University College of Dublin students revealed that 43.2% of students were aware of the usage of cadavers for teaching and research purposes [47]. Most students (95.02%) from the School of Medical and Pharmaceutical Sciences of the University of Genoa, Italy, opined that cadavers are a fundamental source of learning and surgical training purpose [46]. When looking at the study by Jenkin et al., more than 70% of medical students accepted that cadaver donation is essential for medical education [43]. In contrast, in their study among the University of Nairobi Kenya residents, Mwachka et al. found that only 13.9% of undergraduate medical students were aware of the local body donation program [39]. They attributed this low awareness rate to insufficient awareness campaigns and orientation programs for undergraduate medical students. These results suggest that the awareness among medical students is higher in the countries where the source of cadavers is exclusively body donation programs than where the cadaver sources are unclaimed bodies in the majority. In the previous studies done on Indian students, it was found that >60% were aware of the purpose and mode of procurement of bodies in their medical schools [21,38,51,42,44,45,49].

The source of awareness among the students was broadly categorized into three groups: media (television, internet, newspaper, radio), family/friends, and medical persons. In the studies analyzed, it was observed that most of the students were aware of the process of VBD through medical personnel, followed by the media. Interestingly Abbasi et al., in their study, have shown that a significant number of medical students have said the primary source of their awareness was the Department of Anatomy, and the non-medical students attributed their awareness to media. Awareness through family and friends was comparatively lesser [48].

Attitudes toward and the practice of VBD among undergraduate medical students

We broadly divided the attitudes of undergraduate students toward VBD into positive, neutral, and negative. An extensive literature review showed that a student's attitude toward VBD is affected by various factors. Both global and Indian trends show an overall positive attitude among students, but there are some exceptions. Prameela et al., in their study, showed that only 6% of students were willing to donate their bodies for dissection [41] as opposed to 48% of students who showed a willingness toward organ donation. Though there is an overall positive attitude toward VBD, when opinion about donation is categorized into self, by a stranger, or a family member, the study by Mwachaka et al. revealed that 68.1% [39] were opposed to self-body donation and 59.2% recommended donation by strangers. One of the significant factors affecting the attitude is exposure to dissection. Many studies have shown that the students' opinions significantly varied before and after dissection [37, 43, 44,47,50]. After exposure to anatomy dissection, students significantly developed a negative attitude toward pledging VBD themselves and their families. However, Cahill et al. and Perry et al. showed that the attitude toward donation by a stranger did not change significantly even after exposure to dissection [47,52].

**Figure 2:**
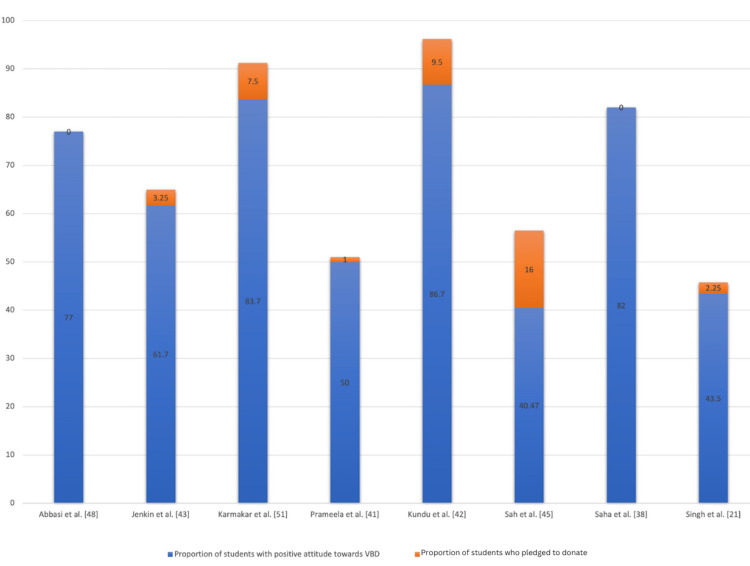
The difference in positive attitudes toward the actual practice of pledging VBD by students in various studies is represented as a percentage distribution VBD: Voluntary body donation

The practice of VBD was assessed in some studies by analyzing how many students pledged VBD. Compared to the high response rates towards willingness to body donation when the actual practice was analyzed, only a few students consented to VBD. The various studies and the number of students who pledged their bodies are given in Figure 2.

Facilitators

The studies included were analyzed, and the facilitators and barriers were grouped into common themes. Facilitator themes were education, gratitude to medical sciences, and motivation. Rokade et al. reported that 90.94% of graduates were ready for VBD compared to 8.9% of those who completed 10th or 12th grade [36]. Sah et al. found that 10.33% of people opined positively about donating their bodies to honor medical science [45]. According to Karmakar et al., 59.8% of respondents were willing to donate their bodies for medical research [51]. According to Singh et al., only seven people (1.86%) were prepared to donate their bodies for educational purposes, while only 5.66% were willing to do so for dissection [21]. 

Prameela et al. identified self-motivation as one of the significant facilitators. In their study, 55.4% of the donors admitted that the motivation for donating their bodies was self-driven [41]. In contrast, Ciliberti et al., in their study, found an altruistic attitude of participants toward VBD [46]. Singh et al. found that more than half the study participants were motivated toward body donation (43.5%) in their study [21].

Lee (&) Lee demonstrated the need for exposure to dissection which increased the respect for body donors [53]. Guo et al. and Lee (&) Lee reported that gratitude ceremony programs conducted in their studies brought about a positive change in attitude among donors [28,53].

Barriers

The willingness of donors toward body donation was high, but the actual practice of donating the bodies was significantly low, as seen in Figure 2. This disproportion could be due to the following barriers: disrespect in cadaver handling, damage to the body, religious beliefs, and discomfort to the family.

Lee (&) Lee found disrespect during cadaver handling as a significant barrier to VBD [53]. Karmakar et al. found that exposure to a dissection hall discouraged individuals against VBD [51]. Rokade et al. found that females had a negative attitude toward VBD compared to males because they thought their bodies would be disrespected during cadaver handling [36]. 

Lee (&) Lee reported damage or wastage of body parts as the second factor that needs to be addressed to change a negative attitude [53]. Several other studies in different parts of India have also raised a similar concern [39,40,41,44,45].

Religious beliefs are a major barrier worldwide toward VBD. Ciliberti et al. pointed out that students who were not religious were 5.9 times more likely to donate their bodies [46]. Most of the studies revealed that religion was a significant barrier [39,40,41,45,46,21,43]. Discomfort to the family members prevented the donors from VBD, as detailed in many studies [38,41,46,21,43]. Mistrust against health services was another significant barrier for VBD, as pointed out by Rokade et al. and Prameela et al [36,41].

Limitations of the original study

Although the questionnaires assessed the overall attitude of the first-year undergraduates toward VBD, they did not assess the actual practice. The cohort was confined to first-year MBBS students only. Comparison with other senior students and postgraduates who had been exposed to dissection and clinical postings for some time now will help analyze the factors responsible for the change of attitudes and practice if any. Analysis of the VBD initiative by faculty and other staff of the Department of Anatomy was not assessed to see if it had any influence on the attitudes and practice of VBD among medical students. The questionnaire scale should have been validated against previously existing scales.

Limitations of the review

This study was done on a relatively limited number of databases for the identification of potentially eligible studies. The assessment of study quality was limited as objective scoring was not done. 

## Conclusions

The present study has shown that the religion of the students and the presence of a known person who donated their body significantly affected their attitudes toward VBD. The original study and the review of relevant literature have shown a positive attitude among medical students toward VBD. However, the change of attitude into actual practice in later life is very low. Based on our observations, we arrived at suggestions to improve the current scenario of students' attitudes toward and practices of VBD. Special training and educational sessions should be organized for undergraduates to increase awareness. A gratitude ceremony for dissected cadavers conducted for students, the dissection staff, and faculty members may help reduce the negative attitude toward VBD after exposure to dissection. Guidelines for the safe and respectful handling of each cadaver used in dissection must be established. Cultivation of an altruistic attitude during the foundation course for students is essential. Media plays a significant role in creating awareness. And so, multimedia should be used effectively to create awareness of the cultural acceptability of VBD.

## Figures and Tables

**Figure 3:**
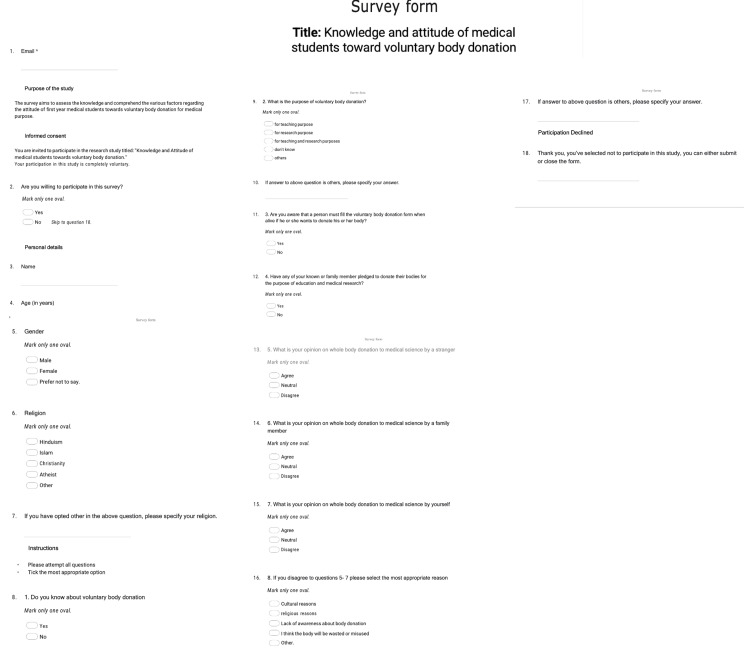
Survey questionnaire for medical students

## Appendices

Appendix A

Appendix B

**Table 4:** Study characteristics of the selected articles included in the review NA: Not available, VBD: Voluntary body donation, BD: Body donation

Authors	Year	Place of study	Keywords	Students involved (UG/PG/Nursing/Paramedical)	Sample size	Age	Gender distribution	Religion	Type of intervention	Knowledge					Attitude			Practice		Facilitators			Barriers				Solutions/sugesstions
										Awareness of procurement	Awareness of the use of cadavers for teaching and research	Source of awareness			Positive	Neutral	Negative	Will they donate?	Will they recommend VBD?	Education	Gratitude to medical sciences	Motivation	Disrespect in cadaver handling/misuse	Damage to the body	Religious beliefs	Discomfort to family	
												Media	Friends and family	Medical persons													
Perry et al. [47]	2009	University College Dublin, Ireland	Anatomical donor program; dissection; death; cadavers; bequeath	First-year graduate	40	52.6% were between 22 and 25 years, 34.2% were between 26 and 29 years, and 7.9% were under 21	51.4% males, 48.6% females	NA	Data obtained via a survey involving the administration of three structured, standardized, and anonymized multi-item questionnaires with five-level Likert scales containing questions designed to measure student responses and attitudes to the idea of whole-body donation to medical science	NA	43.20%	NA	NA	NA	NA	NA	NA	35.1% were strongly supportive. The support reduced as the time increased in medical school	83% by strangers, 54.1% by a family member	NA	NA	NA	NA	NA	NA	NA	Media will play an important role
Rokade et al. [36]	2012	Maharashtra, India	Gross anatomy education; whole-body donation; anatomical donor program; bequest program; attitude to body donation; anatomy dissection; cadaver; undergraduate medical education; India	Medical students	115	NA	NA	NA	Anonymous, prestructured, pretested questionnaire	NA	Aware of BD: 60 males (91.7%) and 50 females (92%)	NA	NA	NA	NA	NA	NA	28 males (46.7%), 19 females (38%)	NA	90.94% of graduates and postgraduates were willing compared to 8.89% of the lower education group (10th/12th)	44% of medical professionals	NA	32 males (27.82%), 34 females (33.66%)	15 males (13.04%), 9 females (8.91%)	Strongly impacted 25 males (21.73%), 20 females (19.8%) agreed	NA	Mistrust toward hospitals negatively influenced VBD
Saha et al. [38]	2015	Kolkata, India	Awareness, cadaver, medical and non-medical population	100 medical students (male: female=70:30), 100 engineering students (male: female=60:40), and 100 doctors (male:female=50:50)	300	18 to 22	70 males, 30 females	Hindu (100%)	Survey	27% were not aware of the pledge form	NA	32.69% motivated by media	48.38% were motivated by family and 8.95% by self	71.80%	62 (62%)	24 (24%)	14 (14%)	NA	NA	NA	NA	NA	70% approximately	30% approximately	All were Hindus. None opted for VBD as religion is a barrier to their willingness	40%	NA
Mwachaka et al. [39]	2016	University of Nairobi (UoN) in Kenya	0	150 first-year UG students, 55 surgical residents (PG)	205	NA	NA	NA	Survey	Yes: 10 (13.9%); no: 62 (86.1%)	NA	NA	NA	NA	16 (10.66%)	7 (12.72%)	49 (89.09%)	16 (10.66%)	NA	NA	NA	NA	NA	NA	7 (17.17%)	NA	NA
Asl et al. [48]	2016	Kashan University of Medical Sciences, Iran	Gross anatomy education; undergraduate education; medical education; body donation; bequest program; personal willingness; medical student; cultural acceptability	331	Medical students: 238 (71.9%); non-medico students: 93 (28%)	17 to 30	Male: 126 (38.1%); Female: 205 (61.9%)	Muslim: 329 (99.4%); Christian: 2 (0.6%)	NA	255 (77.03%)	NA	88 (34.5%)	45 (17.7%)	122 (47.8%)	57 (22.4%)	135 (52.9%)	63 (24.7%)	51 (60.7%)	NA	NA	NA	NA	NA	NA	NA	NA	Cultural acceptability through mass media: 30 (25.6%), respect cadavers: 29 (24.8%), modify religious beliefs: 10 (8.5%)
Kar et al. [40]	2017	Tertiary health care centre of North Bengal, India	Attitude, body bequest program, cadaver, co-donation, willingness	First-year MBBS students, junior doctors, senior doctors, nursing staff, and technicians	69 first-year MBBS	NA	Not given separately for students	Not mentioned	Questionnaire	NA	17 (38.63%) were willing to pledge for medical education and organ donation	42 (23.20%) overall. Not mentioned separately for students	34 (18.78%) overall. Not mentioned separately for students	74 (40.88%) overall. Not mentioned separately for students	63.76% of students	NA	NA	NA	NA	NA	NA	NA	NA	NA	10 (21.73%) overall. Not mentioned separately for students	NA	NA
Prameela et al. [41]	2017	GITAM Dental College and Hospital, Andhra Pradesh,	Awareness, organ and whole body donation, the medical fraternity	Medical students (undergraduates: 3rd & 4th year, graduates, postgraduates)	273 undergraduates (56.4%)	NA	Males: 63, Female: 210	NA	Cross-sectional study with multiple questionnaires	NA	NA	25%	5%	1%	Only 6% toward dissection purpose	NA	NA	46%	NA	NA	NA	Self-motivation: 60%	NA	10% to 15%	15%	15% prevented by family members	Mistrust was a main barrier in 60%
Ghosh et al. [49]	2018	ESI- PGIMSR & ESIC Medical College, Kolkata, West Bengal, India	Body donation; ethics; Unclaimed cadavers; anatomy education; medical students	First-year undergraduate medical students	100	19	Male: 61 (62.2%)l; Female: 37 (37.8%)	NA	Questionnaire	61 (62.2%) did not know the source of cadavers, 85 (86.7%) did not know whom to approach for body donation, 66 (67.5%) did not know about pledging	NA	64 (65.30%)	22 (22.44%)	NA	51 (52%)	9 (9.2%)	38 (38.8%)	NA	NA	NA	NA	NA	NA	NA	NA	NA	Increase awareness, handling bodies with respect
Ciliberti et al. [46]	2018	School of Medical and Pharmaceutical Sciences, University of Genoa, Italy	Postmortem body donation; cadaver; ethics; students’ attitudes; anatomy education; medical education; cadaver lab; unclaimed bodies	Students from all years	1781	19 to 42 years, mean age of 22 years	Male: 208 (44.07%); Female: 264 (55.93%)	Catholic: 266 (56.36%); non-religious: 185 (39.19%); other: 19 (4.03%); Muslim: 1 (0.21%); other: 1 (0.21%)	Both open and close-ended questionnaire	NA	95%	NA	NA	NA	NA	NA	31 (7.09%)	NA	NA	Awareness of the ethical value of body donation	NA	Altruism	NA	NA	Students not holding religious beliefs were 5.9 times more likely to be in favour of donation	Suggested but number not mentioned	NA
Sah et al. [45]	2018	Hind Institute of Medical Sciences, Barabanki and Sitapur, UP, India	Body donation, medical research, willingness, educational qualification	100 medical students (Male: Female=59:41), 100 paramedical students (Male: Female=47:53), 100 nursing students (Male: Female=22:78)	300	243: 17 to 23 years, 45: 24 to 30 years, 7: 31 to 37 years	Males: 128 (42.67%); Females: 172 (57.33%)	Hindus: 278 (92.67%), Muslims: 14 (4.67%), Others: 2%, did not disclose: 2%	Predesigned questionnaire performa	246 (82%) were aware	NA	Internet: 64 (21.33%), newspaper: 50 (16.67%), radio: 35 (11.67%), TV: 42 (14%)	Friends: 200, family members: 77	28 (9.33%)	NA	NA	NA	122 (40.67%) were willing to donate	165 (55%) will recommend donation to family, 199 (66.33%) agreed to convince others to pledge	NA	31 (10.33%)	NA	32 (10.67%)	19 (6.33%)	20 (6.67%)	NA	NA
Biasiutto et al. [50]	2019	Faculty of Medical Sciences, Faculty of Dentistry, National University of Cordoba, Cordoba, Argentina	Anatomy, corpses, cadavers, dissection- room, body donation	First-year students	237	19.27+/-2.25	Males: 84 (35.44%); Females: 150 (63.29%); Did not mention: 3 (1.27%)	127 (55%) Catholic; 88 (38%) no religion; 13 (6%) non-Catholic Christians; 1 Jew	Anonymous surveys with multiple choice and semi-structured answers; 3 surveys: one at the start of the course, one on exposure to cadavers, and the final one just before finishing the course	NA	NA	NA	NA	NA	First survey: 136 (57.38%) positive (60% women); second survey: 114 (49%) positive, 119 (51%) negative; third survey: 119 (52%) positive, 108 (47%) negative	NA	NA	NA	NA	NA	NA	NA	NA	NA	NA	NA	NA
Karmakar et al. [51]	2020	Tripura Medical College and Dr. BRAM Teaching Hospital, Hapania, Tripura, India	Cadaver, death, education, India, mass media	Undergraduate medical students and internees	361	NA	Males: 174 (48.2%); Females: 187 (51.8%)	Hindu: 304 (84.2%); Muslim: 15 (4.2%); Christians: 21 (5.8%); Buddhists: 12 (3.3%), Jain: 1 (0.3%)	Cross-sectional study survey	Yes: 318 (88.1%), No: 43 (11.9%); urban residents, older medicos, and men had better knowledge	NA	50.10%	28.80%	NA	302 (83.7%) younger medicos and men had a positive attitude	36 (10.0%)	23 (6.4%)	27 (7.5%) registered willingness to donate (n= 245 67.86%)	232 (64.3%)	NA	59.60%	NA	People exposed to dissection hall less likely to donate	28.40%	64 (17.7%)	NA	NA
Varalakshmi et al. [44]	2020	Bangalore, India	Body donation, informed consent, autonomy, dignity, confidentiality, post-act benefit	75 medical students (8th and 9th terms), and 75 (final year) engineering students	150	NA	NA	NA	Survey, 5-point Likert scale	57 (76%)	NA	24 (32%)	1 (1.3%)	50 (66.7%)	63 (84%)	7 (9.3%)	5 (6.6%)	NA	NA	NA	NA	NA	NA	37%	NA	NA	NA
Kundu et al. [42]	2021	Chattisgarh, India	Body donation, organ donation, awareness, attitude, medical professionals, donors, tribals	First to final-year undergraduates, n=630 (181 MBBS students and 449 Paramedical staff); MBBS students (each batch 50 students) and all para medical staff and technicians (all graduates) including nursing staff (study sample)	181 MBBS students and 449 paramedical staff	NA	5 (52.49%) MBBS students were male and 86 (47.51%) were female students; 253 (56.35%) male paramedics; 196 (43.65%) female paramedics	NA	Data were obtained by survey, cross-sectional study	165 (91.16%) were aware; the awareness level increased from the first year to the final year of MBBS	NA	44 (24.31%)	17 (9.39%)	109 (60.22%)	165 (91.16%)	NA	NA	84.93%	81.75%	NA	NA	NA	NA	NA	26.52%	NA	NA
Singh et al. [21]	2021	Nepalese Army Institute of Health Sciences, Nepal	Body donation; knowledge; medical students; organ donation	Medical students from the College of Medicine, Basic Science and Clinical Faculties, nursing students from the Faculty of Nursing College, and medical officers from a tertiary care hospital were included in the study	115 medical students, 73 nursing students	223 (58%) were between 18 and 25 years	145 males were between 18 and 25 years, 85 were females in the same age group	369 (91.5%) were Hindus, 20 (5%) were Buddhists	A structured questionnaire was used for the study	360 (90%)	374 (93.5%)	43 (23.5%)	33 (18.9%)	36 (20.6%)	NA	NA	NA	Males: 28 (46.7%)	NA	NA	7 (1.8% )were willing to donate for educational purposes only 5.66% were willing to donate for dissection purposes	174 (43.5%) were motivated nearly half	NA	14%, out of which 3% thought the body would be wasted	14%	14%	NA
Lee (&) Lee [53]	2021	Korea	Cadaver anatomy program, cadaver donation, attitude, intention, nursing student	First-year nursing students	80	NA	Males: 6; Females: 74	Religious: 27 (33.8%), non-religious: 53 (27%); a significant difference according to religion (p<0.001>)	Questionnaire covered general characteristics, attitudes toward cadaver donation, and cadaver donation intention, and the change in the intention for cadaver donation was identified after the end of the four-day practice session	NA	NA	NA	NA	NA	Before exposure: 12.5%; after exposure: 37.5%; total students with a change of attitude: 24 negative than the original i.e., only 9 positive than the original 15	Before exposure: 43 (53.8%)	Before exposure: 27 (33.8%); after exposure: 37 (62.5%); attitude toward cadaver donation had changed more negatively than before	NA	Though they have asked the question they have represented the responses collectively with other responses as positive or negative attitudes toward body donation	NA	NA	Exposure to dissection increases respect to donors; reported as a facilitator but numbers not mentioned	Reported as a factor for change toward a negative attitude	Reported as a factor for change toward a negative attitude	NA	NA	NA
Jenkin et al. [43]	2022	University of Sydney, Australia	Anatomical dissection, attitudes, body donation, cadaver, gross anatomy education, organ donation, postgraduate education, undergraduate education	Undergraduate students. Mathematics (n=133) and Anatomy Experience (n= 172), Health Sciences students (n=279), Medical Sciences students (n=863), Postgraduate Medical and Dentistry students (n=555)	305	Medical science students- 20	Females: 548 (63.5%), Males: 269 (31.2%)	Practice religion 321 (37.6%)	Survey	NA	79%	NA	NA	NA	Willing to donate own body: 243 (28.2%); support family member donation: 599 (69.5%); support donation by a stranger: 717 (83.1%)	Donate own body: 395 (45.8%); support family member donation: 163 (18.9%); support donation by strange: 120 (13.9%)	Donate own body: 225 (26.1%); support family member donation: 100 (11.6%); support donation by a stranger: 26 (3.0%)	Registered donor: 19 (2.2%)	NA	NA	NA	NA	67.3% (overall and not only pertaining to medical sciences students)	NA	17% said religion doesn't allow VBD (overall and not only pertaining to medical sciences students)	30% non-English speaking participants said family will not allow (overall and not only pertaining to medical sciences students)	NA
Guo et al. [28]	2020	Guangzhou, China	Humanistic qualities, medical education, human anatomy, ethics, silent mentor	Third-year medical students	171	20.6 (± 1.0)	72 (42.11%) - males 99 (57.89%) - females	150 atheists, 10 Buddhists 3 Christians, 2 Muslims, and 1 Hindu	Questionnaires	NA	60.82%	NA	NA	NA	NA	NA	NA	NA	NA	NA	NA	NA	NA	NA	NA	NA	Cadaver ceremony to increase respect and awareness
Cahill et al. [52]	2008	University College Dublin, Ireland	Death, anatomical donor program, cadaver dissection, bequeath, anatomy education	First-year medical students	212	18 to 24	Females > Males, numbers NA	Christians	Questionnaires	NA	NA	NA	NA	NA	Stranger: 22.8, 24.5, and 22.5% of respondents to the first, second, and third questionnaires family member: decreased from 31.7% to 14.7% and self from 31.5% to 19.6%	Stranger: neutral; 38.6% of responses to the first questionnaire and 31.4% to the third questionnaire; Family member: 38.9 to 45.4%	Stranger: <1%; family member: increased from 22.9% to 43.1% and self from 23.4% to 40.2%	NA	NA	NA	NA	NA	NA	NA	NA	NA	NA
Anyanwu et al. [37]	2013	University of Nigeria	Organ donation; cadaver dissection; gross anatomy laboratory; psychosocial impacts; anatomy education; altruism; whole body donation	Students and professionals	Students: 780, professionals: 420	NA	Students with dissection experience: 178 (61%) males and 112 (39%) females; students exposed to dissection room without dissection experience: 135 (61%) males and 85 (39%) females; students never exposed to dissection room: 166 (62%) males and 104 (38%) females	NA	Questionnaire	Exposed to dissection: 527 (68%); not exposed to dissection: 277 (44%)	NA	NA	NA	NA	Willingness to donate own body and exposed to dissection: 105 (13%); not exposed to dissection: 70 (17%)	NA	NA	NA	NA	NA	NA	NA	Anxiety related to the mistreatment of cadavers observed in the exposed and dissecting category, religious belief seen in the exposed and non-dissecting category	NA	NA	NA	Strong sanctions should be imposed on the indecent treatment of cadavers by staff and students. The creation of more opportunities in medical school curricula for non-dissecting students who are exposed to the dissection room but do not participate in dissection is advised

## References

[1] Burgess A, Ramsey-Stewart G (2015). Anatomy by whole body dissection: a focus group study of students' learning experience. Adv Med Educ Pract.

[2] Maggio MP, Hariton-Gross K, Gluch J (2012). The use of independent, interactive media for education in dental morphology. J Dent Educ.

[3] Trelease RB (2016). From chalkboard, slides, and paper to e-learning: how computing technologies have transformed anatomical sciences education. Anat Sci Educ.

[4] Bay BH, Ling EA (2007). Teaching of anatomy in the new millennium. Singapore Med J.

[5] Ackerman MJ (2022). The visible human project. Stud Health Technol Inform.

[6] Alasmari WA (2021). Medical students' feedback of applying the virtual dissection table (Anatomage) in learning anatomy: a cross-sectional descriptive study. Adv Med Educ Pract.

[7] Baratz G, Wilson-Delfosse AL, Singelyn BM (2019). Evaluating the Anatomage table compared to cadaveric dissection as a learning modality for gross anatomy. Med Sci Educ.

[8] Bharati AS, N SK, Rani VS (2018). A study on student perception of virtual dissection table (Anatomage) at GSL Medical College, Rajahmundry. Acad Anat Int.

[9] Richardson NS, Zwambag D, McFall K, Andrews DM, Gregory DE (2021). Exploring the utility and student perceptions of synthetic cadavers in an undergraduate human anatomy course. Anat Sci Educ.

[10] McLachlan JC, Bligh J, Bradley P, Searle J (2004). Teaching anatomy without cadavers. Med Educ.

[11] Pawlina W, Lachman N (2004). Dissection in learning and teaching gross anatomy: rebuttal to McLachlan. Anat Rec B New Anat.

[12] Granger NA (2004). Dissection laboratory is vital to medical gross anatomy education. Anat Rec B New Anat.

[13] Ghosh SK (2017). Cadaveric dissection as an educational tool for anatomical sciences in the 21st century. Anat Sci Educ.

[14] Yammine K, Violato C (2015). A meta-analysis of the educational effectiveness of three-dimensional visualization technologies in teaching anatomy. Anat Sci Educ.

[15] Hadie SNH (2018). The application of learning taxonomy in anatomy assessment in medical school. Educ Med J.

[16] Hegazy MA, Mansour KS, Alzyat AM, Mohammad MA, Hegazy AA (2022). A systematic review on normal and abnormal anatomy of coronary arteries. Eur J Anat.

[17] Karunakaran I, Thirumalaikolundusubramanian P, Nalinakumari SD (2017). A preliminary survey of professionalism teaching practices in anatomy education among Indian Medical Colleges. Anat Sci Educ.

[18] Ghazanfar H, Rashid S, Hussain A, Ghazanfar M, Ghazanfar A, Javaid A (2018). Cadaveric dissection a thing of the past? The insight of consultants, fellows, and residents. Cureus.

[19] Memon I (2018). Cadaver dissection is obsolete in medical training! A misinterpreted notion. Med Princ Pract.

[20] Habicht JL, Kiessling C, Winkelmann A (2018). Bodies for anatomy education in medical schools: an overview of the sources of cadavers worldwide. Acad Med.

[21] Singh P, Phuyal N, Khadka S, Gurung M (2021). Knowledge of medical students and faculties of a medical college towards human body and organ donation: a descriptive cross-sectional study. JNMA J Nepal Med Assoc.

[22] Sasi A, Hegde R, Dayal S, Vaz M (2020). 'Life after death - the dead shall teach the living': a qualitative study on the motivations and expectations of body donors, their families, and religious scholars in the South Indian City of Bangalore. Asian Bioeth Rev.

[23] Rath G, Garg K (2006). Inception of cadaver dissection and its relevance in present day scenario of medical education. J Indian Med Assoc.

[24] Oliveira AG, Gonçalves AF, Soares JN (2021). The creation of a body donation program at Federal University of Juiz de Fora in Brazil: academic importance, challenges and donor profile. Anat Cell Biol.

[25] Park HJ, Ahn H, Ki E (2021). Body donation trends in Yonsei University: a statistical analysis of donor records. Anat Cell Biol.

[26] Cornwall J, Poppelwell Z, McManus R (2018). "Why did you really do it?" A mixed-method analysis of the factors underpinning motivations to register as a body donor. Anat Sci Educ.

[27] Agrawal Agrawal (2020). Trends and applications of body donation program in Mahakaushal Region. Natl J Clin Anat.

[28] Guo K, Luo T, Zhou LH (2020). Cultivation of humanistic values in medical education through anatomy pedagogy and gratitude ceremony for body donors. BMC Med Educ.

[29] Jones TW, Lachman N, Pawlina W (2014). Honoring our donors: a survey of memorial ceremonies in United States anatomy programs. Anat Sci Educ.

[30] De Stefano A, Rusciano I, Moretti V, Scavarda A, Green MJ, Wall S, Ratti S (2023). Graphic medicine meets human anatomy: the potential role of comics in raising whole body donation awareness in Italy and beyond. A pilot study. Anat Sci Educ.

[31] Saw A (2018). A new approach to body donation for medical education: the silent mentor programme. Malays Orthop J.

[32] Naidoo N, Al-Sharif GA, Khan R, Azar A, Omer A (2021). In death there is life: perceptions of the university community regarding body donation for educational purposes in the United Arab Emirates. Heliyon.

[33] Jiang J, Zhang M, Meng H (2020). Demographic and motivational factors affecting the whole-body donation programme in Nanjing, China: a cross-sectional survey. BMJ Open.

[34] Ballala K, Shetty A, Malpe SB (2011). Knowledge, attitude, and practices regarding whole body donation among medical professionals in a hospital in India. Anat Sci Educ.

[35] Sehirli US, Saka E, Sarikaya O (2004). Attitudes of Turkish anatomists toward cadaver donation. Clin Anat.

[36] Rokade SA, Gaikawad AP (2012). Body donation in India: social awareness, willingness, and associated factors. Anat Sci Educ.

[37] Anyanwu EG, Obikili EN, Agu AU (2014). The dissection room experience: a factor in the choice of organ and whole body donation--a Nigerian survey. Anat Sci Educ.

[38] Saha A, Sarkar A, Mandal S (2015). Body donation after death: the mental setup of educated people. J Clin Diagn Res.

[39] Mwachaka PM, Mandela P, Saidi H (2016). Repeated exposure to dissection does not influence students' attitudes towards human body donation for anatomy teaching. Anat Res Int.

[40] Kar M, Bhaumik DL, Kar C (2017). Body and organ donation: perception among medical students and medical health professionals in a tertiary care centre. Int J Anat Radiol Surg.

[41] Prameela S, Bhushan N, Sunil T, Kalyan US, Chiang K (2017). Awareness and attitude of medical students towards whole body and organ donation. Int J Adv Res.

[42] Kundu S, Sherke A, Gurudiwan R (2021). Attitudes and myths regarding posthumous whole body bequest and organ donation among medical professionals and health care personnel of tribal Chhattisgarh - a broad questionnaire-based review. Sch J Appl Med Sci.

[43] Jenkin RA, Garrett SA, Keay KA (2023). Altruism in death: attitudes to body and organ donation in Australian students. Anat Sci Educ.

[44] Kl V, Kulkarni U (2020). Life after death: knowledge, attitude and ethical perceptions of medical and engineering students on voluntary body donation. Int J Curr Res Rev.

[45] Sah S, Mishra A, Bhandari K, Chandra N, Choudhary A (2018). A survey of awareness, perception and attitude about whole body donation after death among medical, paramedical and nursing students of hind institute of medical sciences, Barabanki and Sitapur, UP, India. Int J Curr Adv Res.

[46] Ciliberti R, Gulino M, Gazzaniga V (2018). A survey on the knowledge and attitudes of italian medical students toward body donation: ethical and scientific considerations. J Clin Med.

[47] Perry GF, Ettarh RR (2009). Age modulates attitudes to whole body donation among medical students. Anat Sci Educ.

[48] Abbasi Asl J, Nikzad H, Taherian A (2017). Cultural acceptability and personal willingness of Iranian students toward cadaveric donation. Anat Sci Educ.

[49] Kumar Ghosh S, Chakraborty S (2018). Voluntary body donation in India: perceptions of first year medical students. Inv Ed Med.

[50] Biasutto SN, Vargas IEM, Weigandt DM (2019). Reactions of first year medical students in the dissection room, with prosected corpses, and the incidence on own body donation. Rev Argent Anatomía Clínica.

[51] Karmakar N, Chakraborty T, Datta A, Nag K, Das S, Bhattacharjee P (2020). Knowledge, attitude, and practice regarding voluntary whole-body donation among medicos in Northeast India. CHRISMED J Health Res.

[52] Cahill KC, Ettarh RR (2008). Student attitudes to whole body donation are influenced by dissection. Anat Sci Educ.

[53] Lee H-J, Lee S-B (2021). A study on the attitudes of nursing students as regards cadaver donation and change of cadaver donation intention after attending cadaver anatomy program. Ann Romanian Soc Cell Biol.

